# Vaginal microbiome structure in pregnancy and host factors predict preterm birth: Results from the ECHO Cohort

**DOI:** 10.1016/j.annepidem.2025.11.003

**Published:** 2025-11-19

**Authors:** Kimberly S. McKee, Christine M. Bassis, Jonathan Golob, Beatrice Palazzolo, Sarah S. Comstock, Christian Rosas-Salazar, Joseph B. Stanford, Sen Ananda, Thomas O’Connor, James E. Gern, Nigel Paneth, Anne L. Dunlop

**Affiliations:** a Department of Family Medicine, University of Michigan, 1018 Fuller St., Ann Arbor, MI 48104, USA; b Department of Internal Medicine, Division of Infectious Diseases, University of Michigan, 3110, 1500 E Medical Center Dr, Ann Arbor, MI 48109, USA; c Department of Food Science and Human Nutrition, Michigan State University, 469 Wilson Rd #204, East Lansing, MI 48824, USA; d Department of Pediatrics, Vanderbilt University Medical Center, 1211 Medical Center Dr, Nashville, TN 37232, USA; e Department of Family and Preventive Medicine, University of Utah School of Medicine, 375 Chipeta Way A, Salt Lake City, UT 84108, USA; f Departments of Neuroscience and Obstetrics and Gynecology, University of Rochester, 125 Lattimore Rd Suite 200, East Entrance, Rochester, NY 14620, USA; g Department of Pediatrics, University of Wisconsin, 600 Highland Ave, Madison, WI 53792, USA; h Departments of Epidemiology & Biostatistics and Pediatrics & Human Development, Michigan State University, 1200 E Michigan Ave # 145, Lansing, MI 48912, USA; i Department of Gynecology and Obstetrics, Emory University School of Medicine, 201 Dowman Dr, Atlanta, GA 30307, USA

**Keywords:** Preterm birth, Microbiota, Pregnancy

## Abstract

**Purpose:**

The vaginal microbiome is dynamic, typically shifting during pregnancy toward enrichment of Lactobacillus. However, proliferation of Lactobacillus may be absent among women with preterm births (PTBs). We sought to identify robust vaginal microbiota signatures along with host factors that predicted PTB across diverse U.S. cohorts.

**Methods:**

We meta-analyzed 16S rRNA gene amplicon sequence data from the Environmental influences on Child Health Outcomes Cohort. We classified community state types (CSTs) and employed penalized logistic regression models to assess the association between vaginal CST and PTB. We generated supervised random forest models and validated them using a train-and-test approach to identify the most predictive vaginal taxa and host factors.

**Results:**

Of 683 births, 12 % were preterm. Overall, 26 % had a non-L. iners Lactobacillus-dominant CST (I, II, V), 43 % had a L. iners-dominant CST (III), and 30 % had a diverse, non-Lactobacillus-dominant (IV-B, IV-C) CST. Vaginal CST was strongly associated with PTB (adjusted odds ratio [aOR], 3.86, 95 % confidence interval [CI], 1.57–11.3 for diverse, non-Lactobacillus-dominant communities and aOR, 3.03, 95 % CI, 1.25–8.78 for L. iners-dominant compared to L. crispatus-dominant communities). The model with the highest area under the curve (AUC=.77) included Gardnerella vaginalis, age, Prevotella timonensis, and L. crispatus.

**Conclusions:**

Along with host factors, vaginal microbiota could be used for predictive risk scoring for PTB across different U.S. cohorts.

## Background

Preterm birth (PTB), defined as gestational age at birth of < 37 weeks, is a major public health problem and the leading cause of infant morbidity and mortality. Over 500,000 babies are born prematurely each year in the United States (12–13 % of all births), and individuals born preterm are at increased risk of various effects across the life course, ranging from pulmonary and neurodevelopmental effects and impaired vision and hearing at birth to chronic diseases in adulthood [1]. Preterm infants are at increased risk of respiratory distress, hypoglycemia, neonatal death, and developmental delays. PTB is characterized by a collection of phenotypes with several distinct mechanisms [2].

Bacterial vaginosis (BV), the most common lower genital tract infection, is associated with a 1.5- to 3-fold increase in risk for preterm labor [3], and up to 25–40 % of PTBs are considered infection-related. While the reproductive microbiome is dominated by *Lactobacillus* in many, but not all, reproductive-aged women [4], it is a dynamic system, shifting over the course of pregnancy toward increased enrichment of *Lactobacillus* beginning as early as the first trimester [13, 14], which may sustain the pregnancy or promote the transfer of specific microbes to the infant. Thus, pregnant women with term births demonstrate less diversity in their vaginal microbiota than non-pregnant women [8, 10–12], however, this process of *Lactobacillus* proliferation and decreased taxonomic diversity may be absent among women with PTB [11, 12].

Culture-independent methods have demonstrated that the reproductive microbiome is dominated by *Lactobacillus* in many, but not all, reproductive-aged women [4]. While vaginal microbiota can be highly dynamic, the most stable communities have a predominance of *Lactobacillus crispatus*, which is highly associated with community stability—a key marker of community health in the vaginal niche relative to other community types [5]. Host factors such as menstrual cycle day and smoking status can also impact community dynamics [6–9] but have not been well-studied during pregnancy.

Two lines of parallel research suggest the relevance of the interplay between microbiota and the host environment in the context of PTB. First, animal and human gestational studies provide evidence that inflammation influences parturition and that lactic acid produced by *Lactobacillus* can directly affect host immune functions by directly inhibiting pro-inflammatory responses [15]. Second, inflammation also influences host immune responses and is further enhanced during pregnancy in response to increased estrogen [16–18].

Single-site studies have reported disparate associations between vaginal microbiota and PTB. In one case-control study of 40 White women, those with a diverse, polymicrobial community type were more likely to deliver at < 37 weeks, while 75 % of term deliveries had *Lactobacillus*-dominant communities [11]; however, another nested case-control study of 18 cases and 72 controls found no such association [19], and another suggested that microbiota signatures associated with PTB may vary by self-reported race and ethnicity [20]. However, most studies have not included adjustment for host confounders that may drive disparate results and racial differences [21, 22]. Thus, our objective was to leverage data across geographically and demographically diverse cohorts to identify robust vaginal microbiota signatures in pregnancy that, along with host factors, predict PTB across distinct cohorts.

## Methods

### Study population

The Environmental influences on Child Health Outcomes (ECHO) Cohort is a consortium of birth cohorts from across the United States designed to evaluate the impact of *in utero* and early-life exposures on child health outcomes [23]. In addition to well-curated survey and medical record data and biospecimen collections, the present study meta-analyzed 16S rRNA gene amplicon data from ECHO cohorts across a diverse set of sites with available vaginal sequence data. The Atlanta ECHO cohort was recruited between 8- and 14-weeks gestations from prenatal clinics affiliated with two hospitals in Atlanta, GA. The Microbes, Allergy, Asthma, and Pets (MAAP) cohort recruited pregnant women during their second and third trimesters from two hospital systems in the Detroit, MI, metro area, while the Children’s Respiratory and Environmental Workgroup (CREW) ECHO consortium included both the Wisconsin Infant Study Cohort (WISC), drawn from rural medical centers in north-central Wisconsin and the MAAP cohort, drawn from urban metro-Detroit sites. The University of Michigan [U-M] Michigan Archive for Research on Child Health (MARCH) is a population-based cohort recruited from initial prenatal appointments from both the University of Michigan sites and through the remote collection of samples from nine other sites across the state of Michigan. Additional details of the cohorts have been described elsewhere [23–25]. Only women who provided informed consent for providing at least one vaginal swab sample during their pregnancy were included. We analyzed the first sample collected in pregnancy (i.e.7–>28 weeks gestation), and collection protocols varied from the first trimester (MARCH and Atlanta samples) to the third trimester (MAAP, WISC, and non-U-M MARCH samples) (Supplemental Material).

### Clinical and demographic measures

Questionnaires were administered to participants from the first prenatal visit through delivery. Self-identified race and ethnicity were harmonized across sites as a binary variable (i.e. Black or non-Black) due to small cell sizes. Health conditions, parity, delivery information, and antibiotic use were abstracted from the medical record with the exception of the MARCH cohort, from which the following were obtained from the birth certificate: infant’s sex, infant’s birth weight, complications of pregnancy, pre-pregnancy body mass index (BMI), and gestational age. Antibiotic use was harmonized to include any that were taken during pregnancy, and public insurance was used as a proxy for socioeconomic status. We identified spontaneous PTB following spontaneous labor, premature rupture of membranes versus, or indication for an induction from the medical record or birth certificate [26].

### DNA extraction, library preparation, and sequencing

The V4 region of the 16S rRNA gene was amplified using the dual indexing sequencing strategy outlined in the MiSeq standard operating procedure (MARCH) or a modified CTAB buffer protocol (WISC and MAAP) and sequenced using 250 base pair Illumina MiSeq platform using V2 chemistry. The Atlanta cohort underwent amplification of the V3-V4 region of the 16S rRNA gene and was sequenced using 300 base pair paired-end reads. Details of the DNA extraction, library preparation, and sequencing have been summarized elsewhere for MARCH [24], Atlanta [27], and WISC and MAAP [28, 29].

### Bioinformatics

A dual bar-code approach was used for deduplication. We processed raw sequences from all sites together using the DADA2 Workflow for Big Data and dada2 (v.1.5.2) (https://benjjneb.github.io/dada2/bigdata.html) and clustered them into amplicon sequence variants (ASVs). Forward and reverse reads were trimmed using lengths of 255 and 225 base pairs, respectively, and filtered using a minimum quality score of 2. Chimeras were removed as prescribed in the dada2 protocol.

After the removal of one low-quality sample, the total number of reads per sample ranged from 2629–2406,377, with a mean of 63704 (standard deviation [SD]= 63970). We harmonized phylotypes using maximum likelihood amplicon pipeline **(**MaLiAmPi) [30, 31], which is designed to robustly combine 16S amplicon data of various read lengths for meta-analysis using phylogenetic placement of curated full-length 16S vaginal allele references, here sourced from the March of Dimes Database for Preterm Birth Research (https://pretermbirthdb.org/-mod/studydata), mapped to a common tree. Reads from 681 samples were denoised into ASVs, which were then categorized into phylotypes based on a phylogenetic distance of 0.1 to approximate species-level classification. Prior to scaffolding, there was a large amount of variation in global community structure, but phylogenetic scaffolding removed much of the variation by site, as evident in the Uniform Manifold Approximation and Projection ordination plots (Supplemental Material, Figures 1a and 1b).

After quality control filtering and removal of nonbacterial taxa, the relative abundance of a total of 5232 phylotypes was estimated and used to construct Bray-Curtis distances. We classified vaginal samples into community state types (CSTs) using the VAginaL community state typE Nearest CentroId clAssifier (VALENCIA) [32], which uses similarity of samples to reference centroids for classification (Supplemental Material, Figure 2).

### Statistical analysis

We ran Pearson’s chi-squared tests or Fisher’s exact tests with a simulated p-value and Kruskal-Wallis rank sum tests where appropriate to test for significant differences between clinical and demographic characteristics and PTB by cohort. VALENCIA classification resulted in six CSTs (I, II, III, IV-B, IV-C, and V), which we collapsed into the following: non-*iners Lactobacillus* dominant (I, II, and V) (reference), *Lactobacillus iners* (III), and diverse (IV).

We identified potential confounders for inclusion in multivariable models from a set of covariates from the literature that were associated with global community structure using permutational multivariate analysis of variance (PERMANOVA) models (Supplementary Materials) using the *vegan* package in R and the *adonis* function with adjustment for multiple comparisons using a Benjamini and Hochberg false discovery rate criterion of p < 0.05 [33].

After adjustment for all covariates, we reran the final model as a penalized logistic regression, applying Firth’s correction to test the effect of CST category on PTB. In the pooled cohort analysis, we also ran the final model as a mixed-effects model that included a cohort random effect to account for the clustering of participants by site. While a mixed-effects model was fitted, the variance component for “cohort” was estimated to be close to zero with the small cohort random effects. For all multivariable models, we modeled the pooled data and re-ran each model for each cohort individually to test the pooled results’ robustness and generate cohort-specific estimates for comparison.

To rank specific taxa that predicted PTB, we adopted a machine-learning approach utilizing a random forest (RF) classifier for PTB and a test-and-train validation set approach, splitting the data to set aside 20 % for testing (80/20 split). We predicted PTB from three sets of RF models: [1] with all taxa as predictors, [2] with all taxa and host factors, and [3] with the top 20 taxa and PERMANOVA-significant covariates to identify those that were the most predictive of PTB. We ranked specific taxa that contributed the largest amount of homogeneity in the nodes and leaves of the forest trees by estimating the mean decrease in the Gini index and increase in node purity coefficients for categorical predictors. Gini coefficients were estimated each time the tree was split on each feature, with higher values indicating greater discrimination. We generated supervised RF model plots and validated them using receiver operating curves (ROCs) with areas under the curve (AUCs) using “randomForest” based on Breiman’s RF algorithm for classification and regression [34] and the “pROC” package in R. Multidimensional scaling plots of proximity matrix were also used to rank taxa between samples. Models were tuned on the optimal number of variables randomly sampled as candidates at each split (“mtry”) and the optimal number of trees (“ntree”) in R. Default values for the number of variables randomly sampled as candidates at each split were the square root of the number of variables in the model from 5228 taxa. We set the hyperparameter nTree = 500 and tuned with “caret” until the out-of-bag error stopped decreasing.

Each RF model was run twice, first as a complete case analysis and second by using the “na.omit=na.roughfix” setting within randomForest, which imputes the median or mode value for missing covariates. This approach was deemed appropriate given the small amount of missingness in the metadata, with the exception of antibiotic use, as it was not missing completely at random. To compare prediction models for PTB, we generated ROCs for each of three RF models and compared AUCs for each. All statistical analyses were conducted using RStudio (R version 4.2.3) [35].

## Results

We analyzed vaginal 16S sequence data from pregnant participants across several geographically distinct areas of the United States. After we removed data from 2 participants with deliveries > 42 weeks gestational age, the sample size was N = 677 (Table 1). Approximately 12 % of women had hypertension in pregnancy, 3.7 % were diagnosed with gestational diabetes, and 12 % had a PTB, of which 75 % were spontaneous (Table 1). The mean age was 28 years (SD 5.4), 43 % had a BMI ≥ 18.5–< 25 kg/m^2^, 42 % were nulliparous, and 12 % smoked in pregnancy. Based on self-report, 63 % of participants were Black, 37 % were non-Black, and 1.9 % were Latinx. Antibiotic use in pregnancy was common (38 %) in all trimesters of pregnancy. No more than 0.6 % of the covariate data were missing for the pooled analyses, except for antibiotic use, which was missing from one cohort.

There were significant differences between term births and PTBs according to self-reported race, with 84 % of PTBs resulting from pregnancies among self-identified Black women (p < 0.001). Other significant differences between outcomes were based on maternal education (42 % of PTBs occurred among participants with high school/GED or less; p < 0.001), receipt of public insurance (85 % among those with PTB; p < 0.001), and vaginal CST (non-*iners Lactobacillus*-dominant communities were less common [6.8 %] among those with PTBs compared to term births [29 %], while diverse communities were more common [44 %] among those with PTBs than among those with term births [29 %]; p < 0.001) (Table 1). Demographics also varied by cohort, most notably for race since the Atlanta cohort, by design, was composed entirely of Black individuals, while the subset of the MARCH cohort with vaginal microbiome data was 13% Black and that of WISC was 10% Black (Supplementary Materials).

**Fig. 1.**
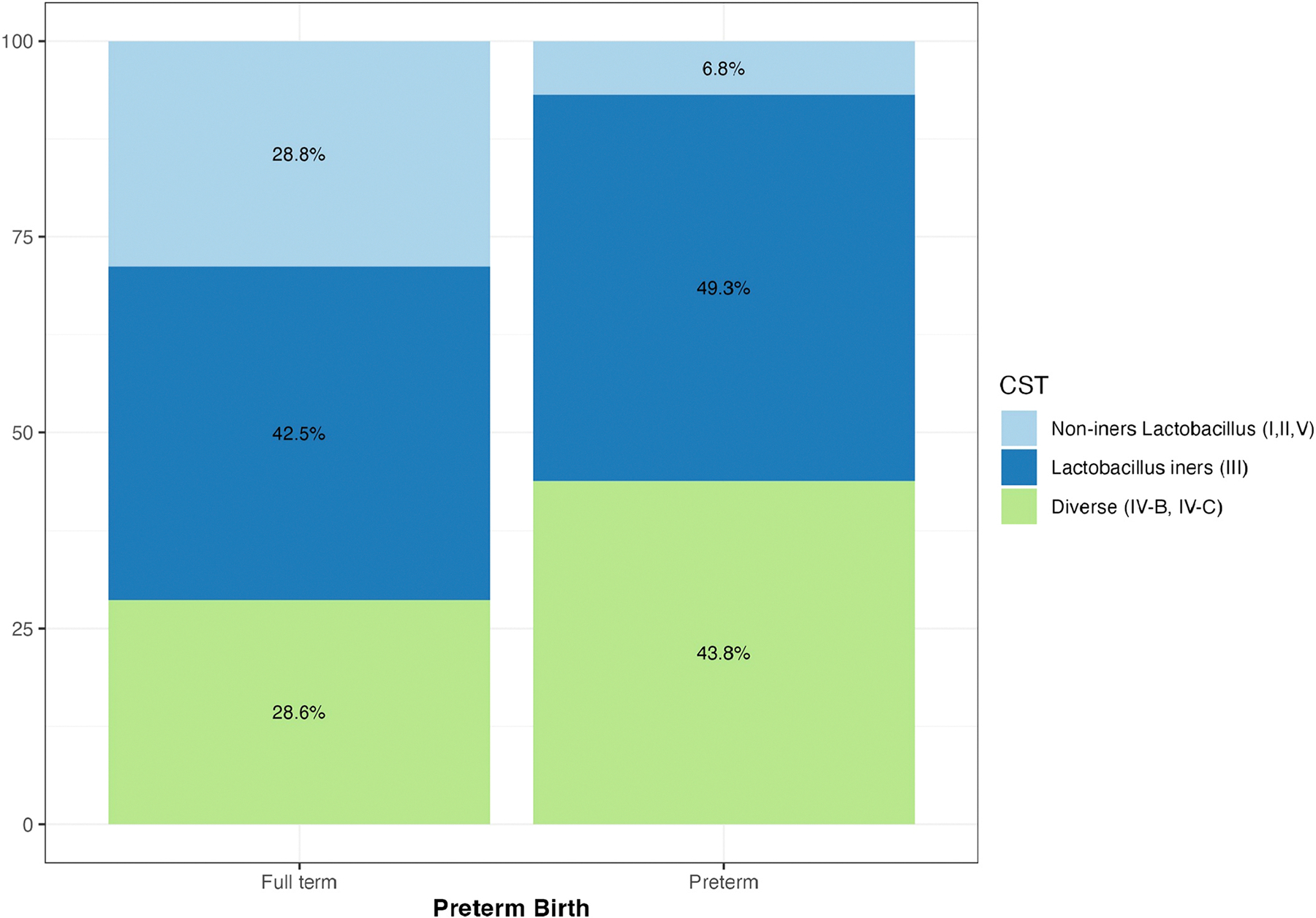
Prevalence of VAginaL community state typE Nearest CentroId clAssifier (VALENCIA) community state types (CSTs) by preterm and term births.

Four CSTs were *Lactobacillus* dominant: CST I, *L. crispatus*; CST II, *L. gasseri*; CST III, *L. iners*; and CST V, *L. jenseni*. The remainder were diverse, polymicrobial communities: IV-B, characterized by high *G. vaginalis*, low *Candidatus Lachnocurva vaginae* (formerly known as *BVAB1*), and moderate *Atopobium vaginae* relative abundances, and IV-C, characterized by a diverse array of facultative and anaerobic bacteria and low relative abundances of *Lactobacillus spp., G. vaginalis, A. vaginae*, and *Ca. L. vaginae* (Supplementary Materials). The distributions of CSTs varied by cohort, and the prevalence significantly varied by PTB. Among PTBs, the most common was CST III (49.3% compared to 42.5% among term births) followed by CST IV-B (43.8% compared to 28.6% among term births) (Figure 1).

**Fig. 2.**
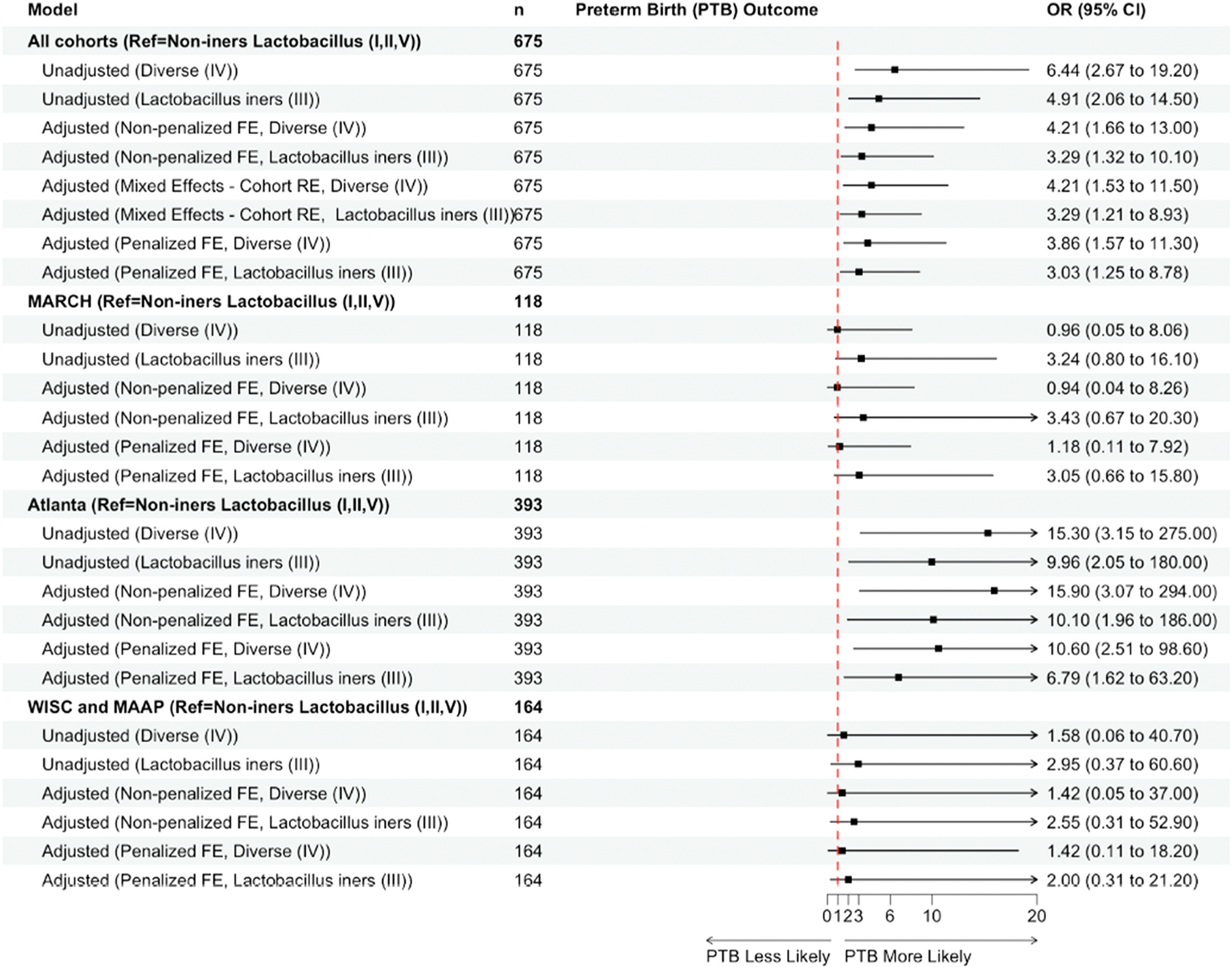
The association of vaginal community state types and preterm birth in pooled analyses (top) and by cohort (bottom). Abbreviations: CI, confidence interval; FE, fixed effects; OR, odds ratio; MAAP, Microbes, Allergy, Asthma, and Pets; MARCH, Michigan Archive for Research on Child Health; PTB, preterm birth; RE, random effects; WISC, Wisconsin Infant Study Cohort.

In our pooled analyses, compared to *L. crispatus-*dominant communities, both diverse *non-Lactobacillus*-dominant communities (odds ratio [OR], 6.44; 95% confidence interval [CI], 2.67–19.20) and *L. iners*-dominant communities (OR, 4.91; 95% CI, 2.06–14.50) were associated with PTB (Table 2). Vaginal CSTs remained significantly associated with PTB even after adjustment for self-reported race, maternal age, level of educational attainment, and parity for diverse, non-*Lactobacillus-*dominant communities (adjusted OR [aOR], 3.86; 95% CI, 1.57–11.3) and *L. iners*-dominant communities (aOR, 3.03; 95% CI, 1.25–8.78) compared to *L. crispatus-*dominant communities (Figure 2). In cohort-specific analyses, the associations between diverse *non-Lactobacillus*-dominant communities and *L. iners*-dominant communities and preterm birth were not significant in the smaller MARCH, WISC, and MAAP cohorts although they were robustly associated in the Atlanta cohort (Figure 2).

**Fig. 3.**
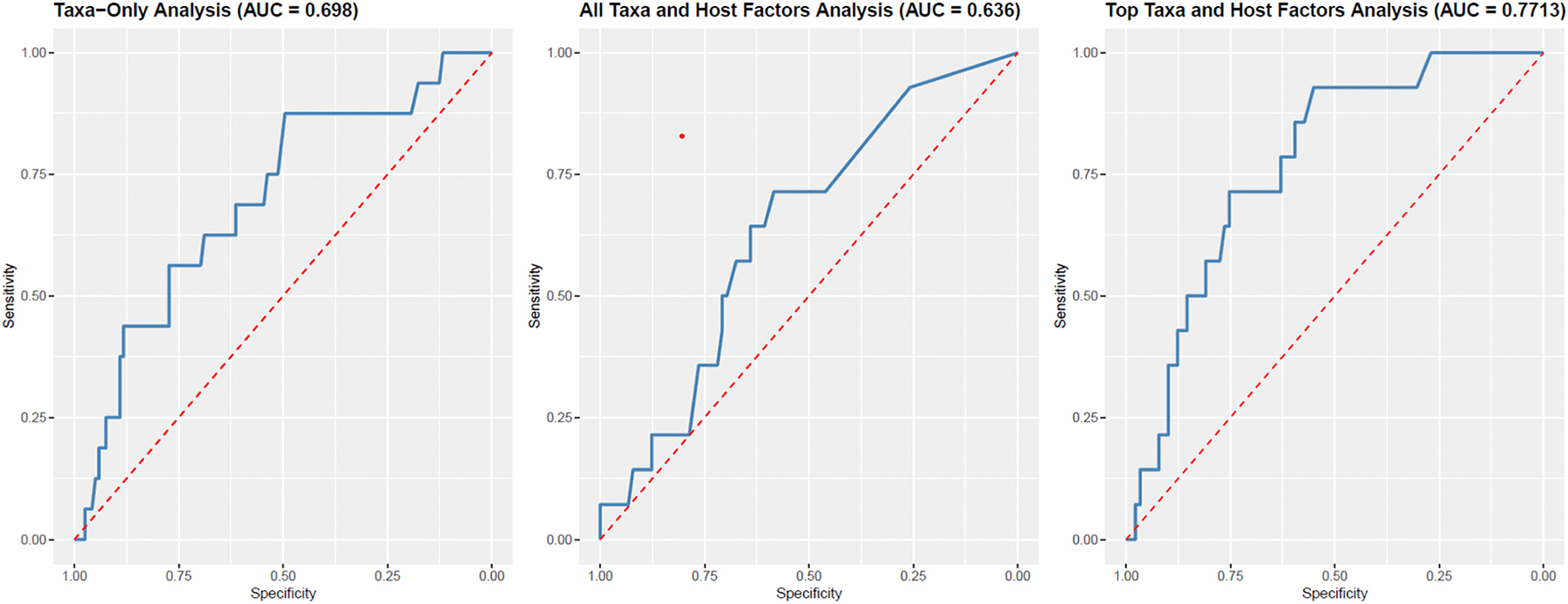
Comparison of receiver operating curves of taxa-only (left), all taxa and host factor (middle), and top-ranked taxa and host factor random forest models. Abbreviations: AUC, area under the curve.

**Fig. 4.**
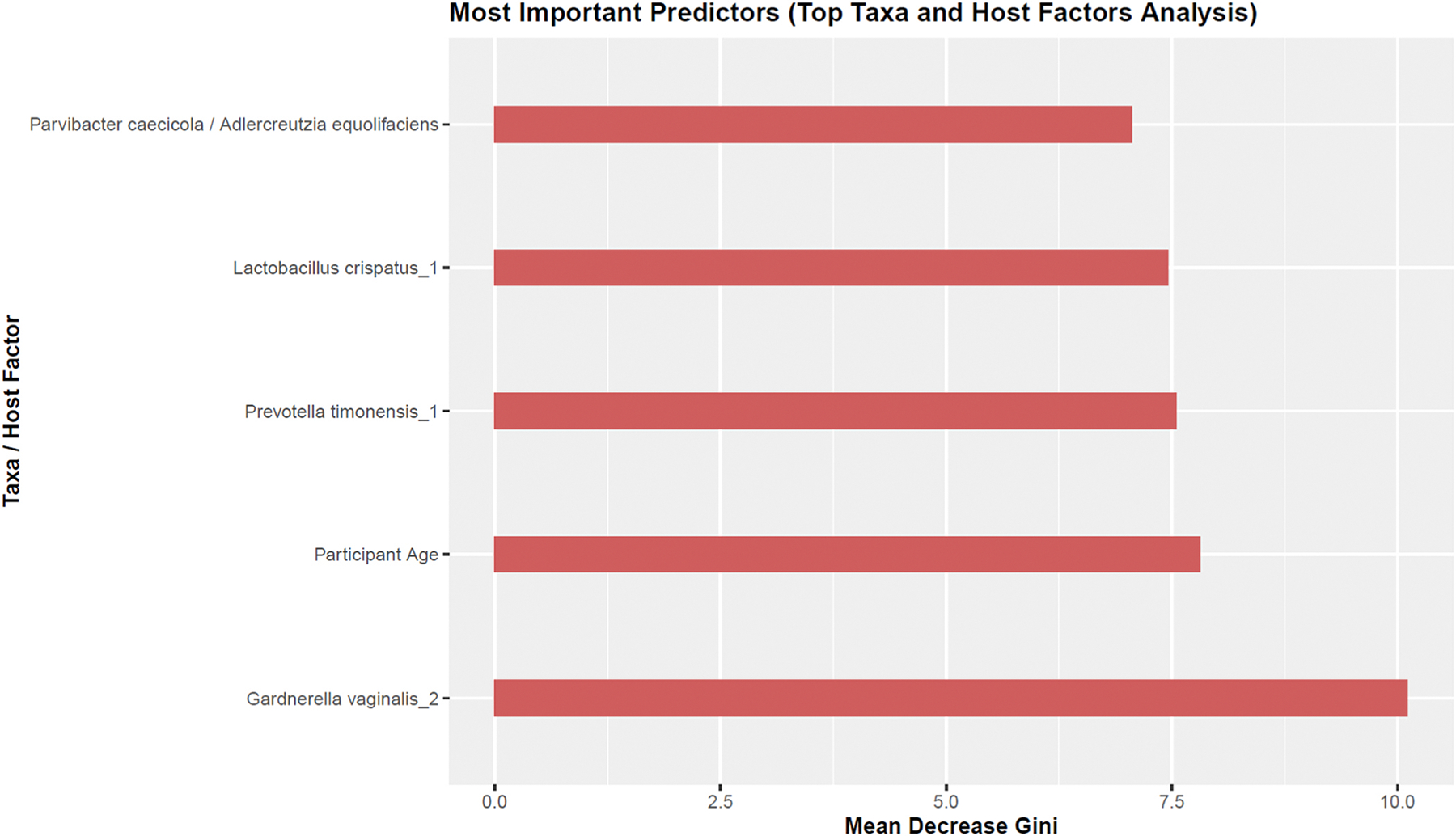
The taxonomic and host features most predictive of preterm birth from random forest models. Abbreviations: AUC, area under the curve.

Using RF models, we identified taxonomic and host factor predictors of PTB. The highest AUC was 0.77 for the RF model with top-ranked vaginal taxa and maternal factors, which was the most informative, compared with AUCs of 0.70 for the all-taxa model and 0.64 for the combined all-taxa and host factor model (Figure 3). The most predictive taxonomic features included Gardnerella vaginalis and Prevotella across all machine learning models (Supplemental Material, Figures 2 and 3). The top five features of the most parsimonious model included *Gardnerella vaginalis,* participant age, *Prevotella timonensis*, and *L. crispatus* (Figure 4).

In sensitivity analyses, CST was strongly associated with spontaneous PTB but not with idiopathic PTB (Supplementary Materials). We also tested whether adjustment for antibiotic use in pregnancy, which was not available for WISC participants, affected the results in our pooled analyses, but the results were qualitatively similar to our main results (Supplementary Materials).

## Discussion

By leveraging vaginal microbiota and extensive host metadata from the ECHO cohort, we confirmed that vaginal microbiota CST was robustly predictive of PTB, and we identified taxonomic and host features in pregnancy that can predict the risk of PTB. Our results are consistent with those of smaller single-site studies [36, 37] and others [11, 38] that associated the risk of PTB with depletion of *L. crispatus*, a key indicator of community stability and pathogen resistance. Our study also demonstrates that a lack of non-*iners Lactobacillus* dominance and polymicrobial states is linked to BV and that obligate anaerobes are predictive of PTB. Dunlop et al. showed similar results among African American women, and Elovitz et al. showed that these factors combined with vaginal inflammation were predictive of PTB [21, 27, 39–41].

The most predictive features of PTB included *Gardnerella vaginalis* and *Prevotella* across all models, which confirm and extend specific taxonomic signatures associated with PTB in single-site studies. *Gardnerella vaginalis* is associated with BV and often co-occurs with *L. iners*, a marker of instability of the vaginal microbiome structure and pregnancy complications. Both the dominant features of CSTs and the relative abundance of pathogens may be key facets of the microbial architecture that is predictive of PTB.

We observed some heterogeneity in results by cohort, but some degree was expected due to the large inter-personal variation in the microbiome in general. However, the differences and their implications for health in the ECHO cohorts were arguably smaller in magnitude with more robust findings across cohorts than previous studies [21]. We would also underscore that while there were differences across the cohorts likely from difference in prevalence of individual CSTs across cohorts, there were consistent community features with regard to clinical markers of health.

In previous work examining the effect of host factors on vaginal microbiome structure in pregnancy, we observed that social factors, and maternal education in particular, were more robustly associated with microbial composition than clinical factors. In the present study, we adjusted our preterm birth estimates for several of these host factors for which many previous studies have not adequately accounted. They also underscore the importance of including host metadata, especially when meta-analyzing vaginal 16S gene amplicon data across cohorts.

Our results are also consistent with previous single-site studies that have used machine-learning approaches to rank features predictive of PTB in terms of their physiological effects and function, but they extend these results by including host factors. Whereas previous studies have generated low to modest AUC scores of 0.28–0.69 for vaginal microbiota-associated features [42], those studies have not included host features to enhance the prediction of PTB. The addition of well-curated metadata on ECHO pregnancies in our machine-learning models improved the prediction of PTB to an AUC of 0.77, indicating that the inclusion of clinical and demographic factors is important for future research.

A major strength of the study was the inclusion of well-curated metadata from ECHO and the large size and diversity of the ECHO cohorts. We used a novel tool to pool 16S gene amplicon data using phylogenetic mapping rather than estimating their effects separately, which increased the power and precision of our results. One limitation was that the cohorts in our study had different sample collection protocols, including varied gestational ages at sample collection which occurred prior to the conception of our study. The vaginal microbiota in pregnancies that result in term births are highly stable across gestation [14], but for some pregnancies that result in preterm birth, there can be more instability in the vaginal niche, and the first compared to third trimester samples may vary [19]. Furthermore, the WISC and MAAP samples were recto-vaginal samples collected at group B strep screenings, which may have attenuated some of the microbiome differences related to PTB. In addition, limited data availability for some factors (measures of diet, cohabitation/marital status, and douching practices) precluded or limited the power of some comparisons [43, 44] that may have resulted in residual confounding. While we were not able to harmonize the gestational week at sample collection, we aligned the sequence data to the first collection in pregnancy.

In conclusion, our study advances the understanding of vaginal microbiome associations with PTB by analyzing a large, diverse sample from four birth cohorts. These results help establish relationships among host characteristics, vaginal microbiota, and PTB and may be useful for identifying prediction targets.

## Supplementary Material

1

Supplementary data associated with this article can be found in the online version at doi:10.1016/j.annepidem.2025.11.003.

## Figures and Tables

**Table 1:** Clinical and Demographic Characteristics by Preterm Birth, Reported as n (%).

Variable	Overall, N = 677	Full term, N = 604	Preterm, N = 73	p-value

CST				< 0.001
Non-*iners Lactobacillus* (I, II, V)	179 (26 %)	174 (29 %)	5 (6.8 %)	
*Lactobacillus iners* (III)	293 (43 %)	257 (43 %)	36 (49 %)	
Diverse (IV-B, IV-C)	205 (30 %)	173 (29 %)	32 (44 %)	
Cohort				< 0.001
Atlanta	393 (58 %)	335 (55 %)	58 (79 %)	
MARCH	120 (18 %)	110 (18 %)	10 (14 %)	
WISC	164 (24 %)	159 (26 %)	5 (6.8 %)	
Self-reported Race				< 0.001
Black	425 (63 %)	364 (61 %)	61 (84 %)	
Non-Black	248 (37 %)	236 (39 %)	12 (16 %)	
(Missing)	4	4	0	
Hispanic				> 0.9
No	655 (98 %)	583 (98 %)	72 (99 %)	
Yes	13 (1.9 %)	12 (2.0 %)	1 (1.4 %)	
(Missing)	9	9	0	
Maternal Age				0.006
Mean (SD)	27.7 (5.4)	27.9 (5.4)	26.1 (5.2)	
(Missing)	1	0	1	
Education				< 0.001
BA or Higher	247 (37 %)	235 (39 %)	12 (17 %)	
HS/GED	171 (25 %)	141 (23 %)	30 (42 %)	
Less than HS	63 (9.3 %)	54 (9.0 %)	9 (12 %)	
Some College/Assoc.	194 (29 %)	173 (29 %)	21 (29 %)	
(Missing)	2	1	1	
Public Insurance				< 0.001
No	224 (40 %)	214 (44 %)	10 (15 %)	
Yes	333 (60 %)	275 (56 %)	58 (85 %)	
(Missing)	120	115	5	
Antibiotics Ever in Pregnancy				0.7
No	318 (62 %)	277 (62 %)	41 (60 %)	
Yes	194 (38 %)	167 (38 %)	27 (40 %)	
(Missing)	165	160	5	
Antibiotics in First				> 0.9
Trimester				
No	423 (83 %)	367 (83 %)	56 (82 %)	
Yes	89 (17 %)	77 (17 %)	12 (18 %)	
(Missing)	165	160	5	
Antibiotics in Second				0.7
Trimester				
No	430 (84 %)	374 (84 %)	56 (82 %)	
Yes	82 (16 %)	70 (16 %)	12 (18 %)	
(Missing)	165	160	5	
Antibiotics in Third Trimester				0.7
No	451 (88 %)	392 (88 %)	59 (87 %)	
Yes	61 (12 %)	52 (12 %)	9 (13 %)	
(Missing)	165	160	5	
Birth Sex				0.045
Female	344 (51 %)	315 (52 %)	29 (40 %)	
Male	333 (49 %)	289 (48 %)	44 (60 %)	
Smoking in Pregnancy				> 0.9
No	585 (88 %)	521 (88 %)	64 (88 %)	
Yes	82 (12 %)	73 (12 %)	9 (12 %)	
(Missing)	10	10	0	
Gestational Diabetes				> 0.9
No	600 (96 %)	529 (96 %)	71 (97 %)	
Yes	23 (3.7 %)	21 (3.8 %)	2 (2.7 %)	
(Missing)	54	54	0	
Hypertension				0.06
No	546 (88 %)	487 (89 %)	59 (81 %)	
Yes	77 (12 %)	63 (11 %)	14 (19 %)	
(Missing)	54	54	0	
BMI Category				0.067
Normal (≥18.5-<25)	270 (43 %)	243 (44 %)	27 (38 %)	
Obese (≥30)	191 (31 %)	174 (31 %)	17 (24 %)	
Overweight (≥25-<30)	141 (23 %)	119 (22 %)	22 (31 %)	
Underweight (<18.5)	22 (3.5 %)	17 (3.1 %)	5 (7.0 %)	
(Missing)	53	51	2	
Parity Category				0.047
1	221 (33 %)	203 (34 %)	18 (25 %)	
2	101 (15 %)	85 (14 %)	16 (22 %)	
3 +	69 (10 %)	57 (9.4 %)	12 (16 %)	
No prior	286 (42 %)	259 (43 %)	27 (37 %)	
Spontaneous Preterm Birth				> 0.9
No	17 (25 %)	-	17 (25 %)	
Yes	51 (75 %)	-	51 (75 %)	
(Missing)	5*	-	5*	

Abbreviations: BA, Bachelor of Arts; BMI, body mass index; CST, community state type; GED, General Educational Development; HS, high school; MARCH, Michigan Archive for Research on Child Health; SD, standard deviation; WISC, Wisconsin Infant Study Cohort.

Analyses included Pearson’s chi-squared test, Fisher’s exact test, Wilcoxon rank sum test, and Fisher’s exact test for count data with simulated p-value (based on 2000 replicates)

* Not enough data to derive spontaneous and induced preterm birth for WISC

**Table 2:** Vaginal Community State Types Associated with Preterm Birth.

	Model 1 (n = 675)			Model 2 (n = 675)			Model 4 (n = 675)			Final Model – Penalized (n = 511)		

Variable	OR	95 % CI	p-value	OR	95 % CI	p-value	OR	95 % CI	p-value	OR	95 % CI	p-value
**CST**												
Non-*iners Lactobacillus* (I, II, V)	—	—		—	—		—	—		—	—	
Diverse (IV)	6.44	2.67, 19.2	< 0.001	4.81	1.95, 14.5	0.002	4.31	1.70, 13.3	0.004	5.16	1.88, 17.4	< 0.001
*Lactobacillus iners* (III)	4.91	2.06, 14.5	0.001	3.85	1.59, 11.5	0.006	3.45	1.38, 10.5	0.015	3.76	1.39, 12.6	0.008
**Non-Black**												
No				—	—		—	—		—	—	
Yes				0.4	0.20, 0.75	0.007	0.5	0.23, 1.07	0.082	0.67	0.25, 1.62	0.4
**Maternal Age**							1.01	0.95, 1.07	0.8	1	0.94, 1.07	> 0.9
**Education Level**												
BA or Higher							—	—		—	—	
HS/GED							1.95	0.84, 4.70	0.13	1.5	0.61, 3.84	0.4
Less than HS							1.4	0.47, 4.07	0.5	1.09	0.35, 3.34	0.9
Some College/Assoc.							1.33	0.59, 3.06	0.5	0.99	0.41, 2.48	> 0.9
**Parity Category**												
> =1										—	—	
0										0.76	0.43, 1.32	0.3
**Antibiotics Ever in Pregnancy**												
None										—	—	
Yes										0.75	0.43, 1.31	0.3

Abbreviations: BA, Bachelor of Arts; CI, confidence interval; CST, community state type; GED, General Educational Development; HS, high school; OR, odds ratio.

## Data Availability

The unprocessed 16S rRNA gene amplicon data are publicly available for the Atlanta cohort (https://www.ncbi.nlm.nih.gov/sra, PRJNA725416) and WISC (European Nucleotide Archive European Nucleotide Archive [ENA]: PRJEB46659). Select de-identified data from the ECHO Program are available through NICHD’s Data and Specimen Hub (DASH). Information on study data not available on DASH, such as some Indigenous datasets, can be found on the ECHO study DASH webpage. Code for the study can be accessed publicly (https://github.com/kimckee-umich/Vaginal-Microbiome-Structure-in-Pregnancy-Results-from-the-ECHO-Cohorts).

## Abbreviations:

ASV: amplicon sequence variants
AUC: area under the curve
BMI: body mass index
BV: bacterial vaginosis
CST: community state type
ECHO: Environmental influences on Child Health Outcomes
MAAP: Microbes, Allergy, Asthma, and Pets
MaLiAmPi: maximum likelihood amplicon pipeline
MARCH: Michigan Archive for Research on Child Health
PERMANOVA: permutational multivariate analysis of variance
PTB: preterm birth
RF: random forest
ROC: receiver operating curve
SD: standard deviation
U-M: University of Michigan
VALENCIA: VAginaL community state typE Nearest CentroId clAssifier
WISC: Wisconsin Infant Study Cohort
