# Supplementary material for: Vaginal microbiome structure in pregnancy and host factors predict preterm birth: Results from the ECHO Cohort
Source: Ann Epidemiol. Author manuscript; Available in PMC 2026 Jul 17. (PMC13378605; doi:10.1016/j.annepidem.2025.11.003)

**Vaginal microbiome structure in pregnancy and host factors predict preterm birth: Results from the ECHO Cohort**

**Supplementary Material**

**ECHO Cohort Study Populations**

The Michigan Archive for Research on Child Health (MARCH) is an ongoing population-based pregnancy and birth cohort recruited from prenatal clinical sites. In the Atlanta cohort, participants were recruited from prenatal clinics affiliated with two hospitals in Atlanta, GA, at 8–14 weeks gestation. The MAAP cohort included pregnant women during their second and third trimesters from two hospital systems in metro-Detroit to understand how exposures in early life modify risk for asthma. The WISC identified eligible pregnant women using electronic health records from a hospital and 10 rural medical centers throughout north-central Wisconsin. The MARCH cohort recruited pregnant women from their first prenatal appointment from 10 sites, although most of the vaginal samples were recruited from two clinics at U-M. The Atlanta African American Maternal-Child Cohort (hereafter referred to as the Atlanta cohort) enrolled African American women from private and public prenatal clinics in Atlanta, GA. The Wisconsin Infant Study Cohort (WISC) includes women and their children from rural and small town Wisconsin, while the Microbes, Allergy, Asthma, and Pets (MAAP) population is from urban Detroit sites.

**Vaginal Sample Collection**

At the MARCH U-M site, vaginal dual-headed dry swabs (Starplex™ Scientific S09D, Fisher Scientific) were self-collected in the prenatal clinics, and immediately upon collection, AllProtect (Qiagen) was added to the swabs prior to archival storage. At the remaining nine MARCH sites, vaginal dual-headed dry swabs (Starplex™ Scientific S09D, Fisher Scientific) were self-collected and mailed to the laboratory. Similarly, in the Atlanta cohort, vaginal swabs were self-collected using the Sterile Catch-All Sample Swab (Epicentre) and placed immediately in MoBio bead tubes prior to archival storage. At the WISC and MAAP sites**,** vaginal/rectal swabs (Epicentre Catch-All™) were collected by a clinical provider within 6 weeks of delivery at the time of Group B Streptococcus screening and stored in RNAlater at 4° C until archival storage.

**Legends for Supplementary Figures.**

Supplementary Figure 1a. Principal coordinates (PCo) analysis of Bray–Curtis distances between samples based on amplicon sequence variants prior to MaLiAmPi processing. CREW, Children’s Respiratory Research and the Environment Workgroup; WISC Wisconsin Infant Study Cohort; MAAP, Microbes, Allergy, Asthma, and Pets; MARCH, Michigan Archive for Research on Child Health; U-M, University of Michigan.

Supplementary Figure 1b. Phylotypes demonstrating that using phylogenetic placement of amplicon sequence variants on a reference tree removed a large degree of variation by site. CREW, Children’s Respiratory Research and the Environment Workgroup; WISC Wisconsin Infant Study Cohort; MAAP, Microbes, Allergy, Asthma, and Pets; MARCH, Michigan Archive for Research on Child Health; U-M, University of Michigan; WISC, Wisconsin Infant Study Cohort

Supplementary Figure 2. Taxonomic composition of VAginaL community state typE Nearest CentroId classifier (VALENCIA) community state types (CSTs).

Supplementary Figure 3. Most predictive features of all taxa and host factor random forest models of preterm birth.

Supplementary Figure 4. Most predictive features of the top taxa and host factor random forest models of preterm birth.

Supplementary Figure 1a


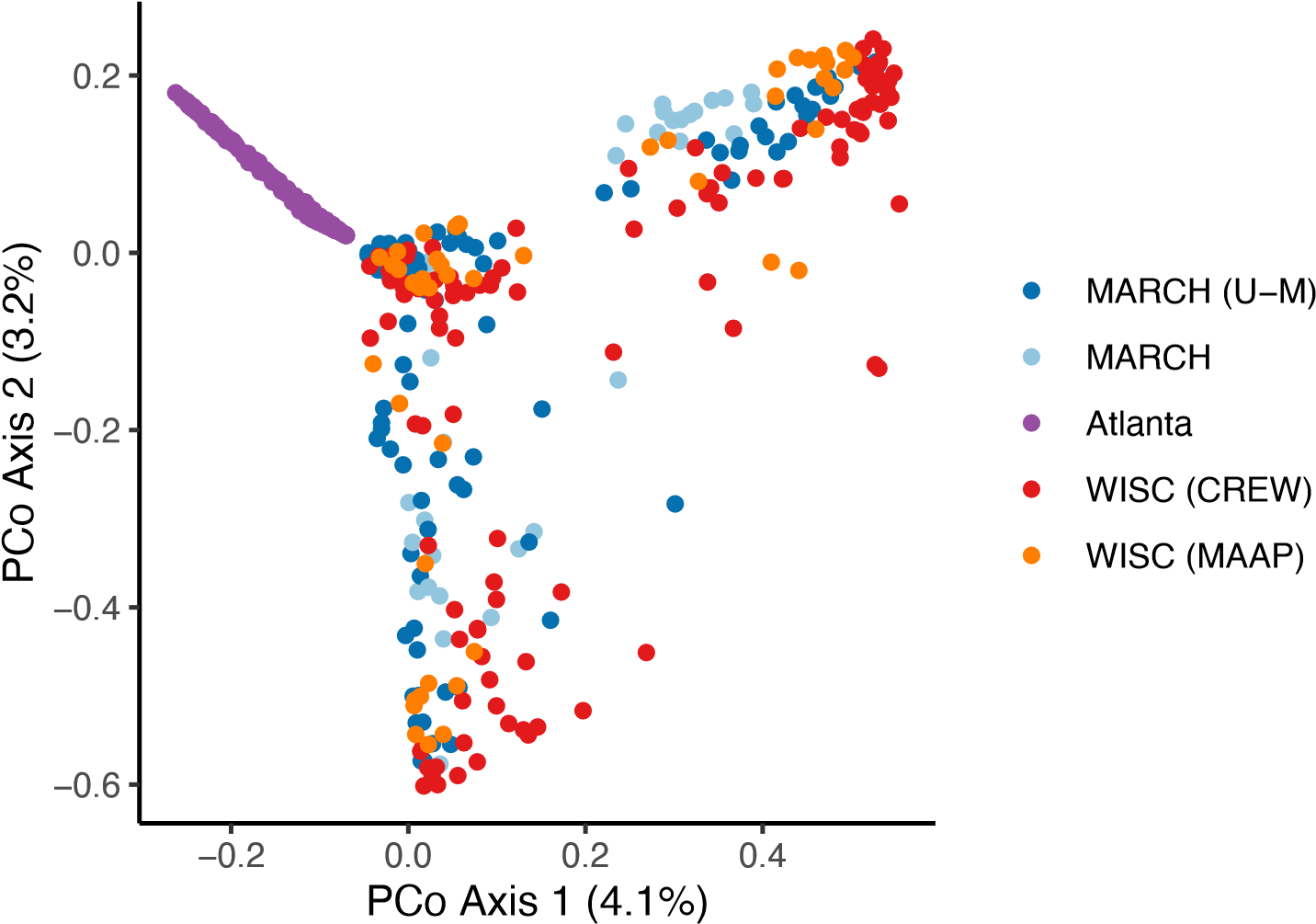


Supplementary Figure 1b


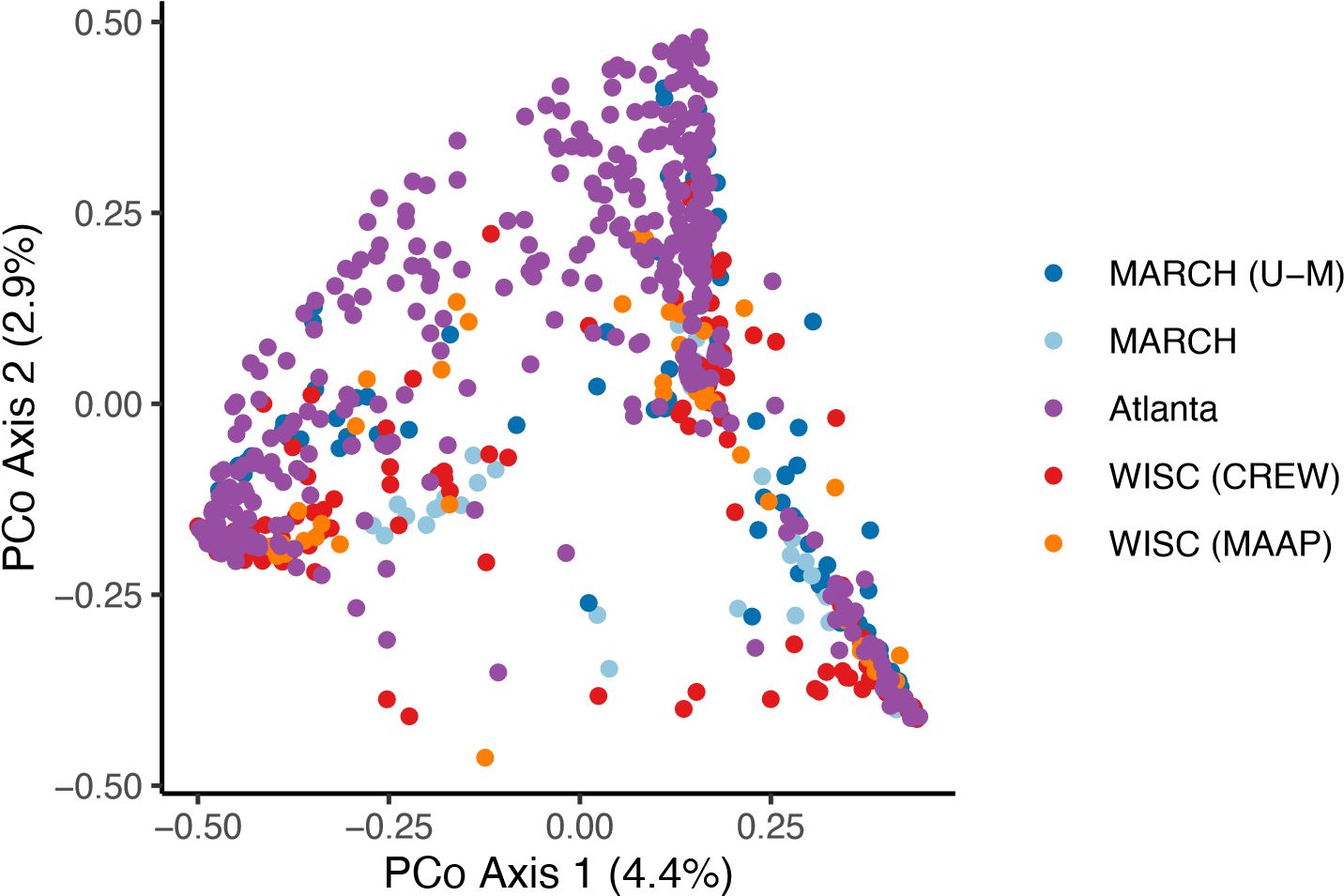


Supplementary Figure 2


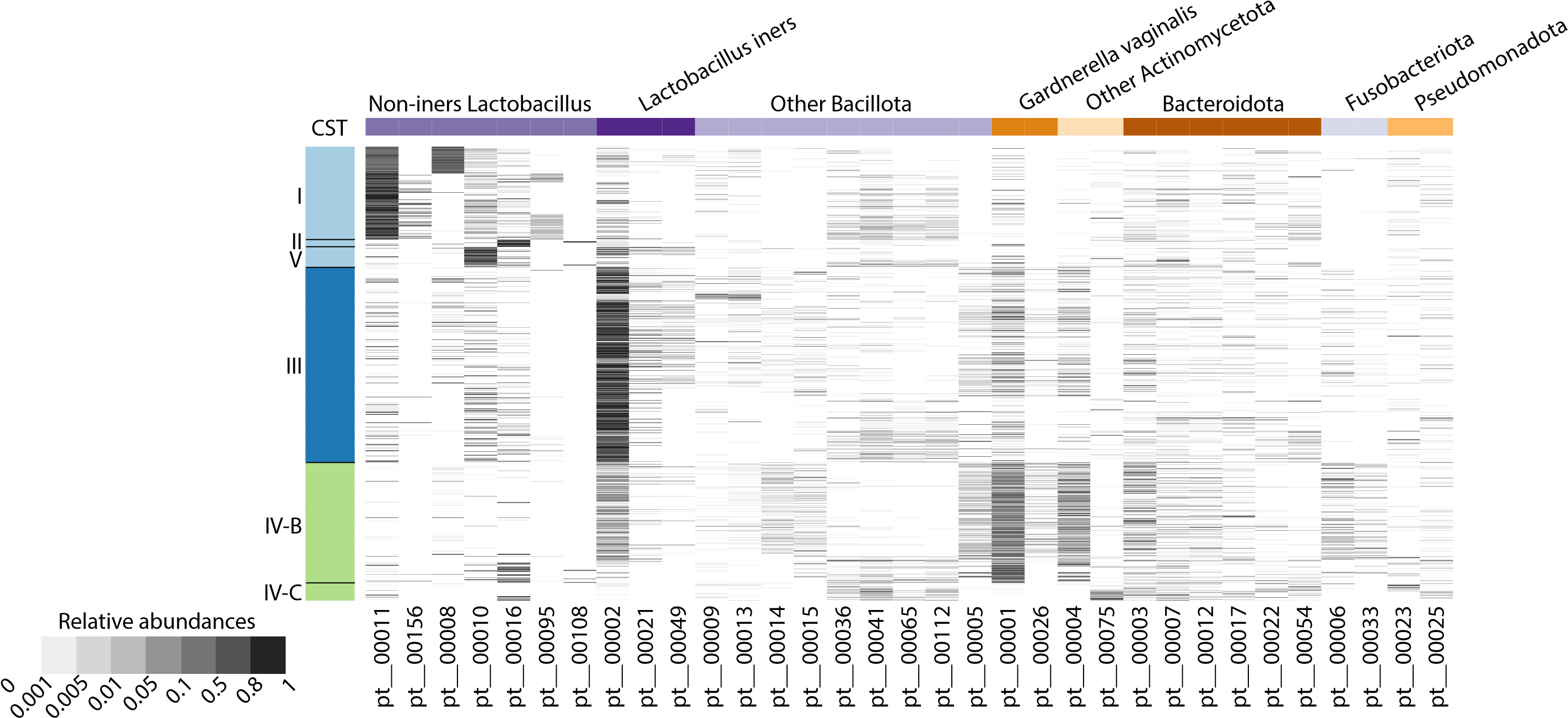


Supplementary Figure 3


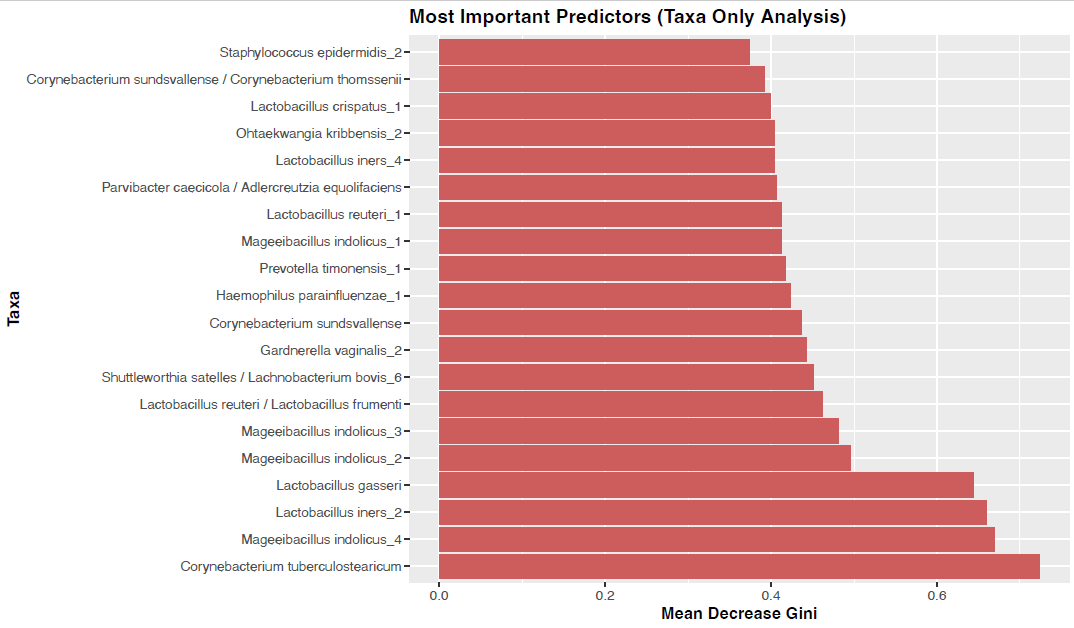


Supplementary Figure 4


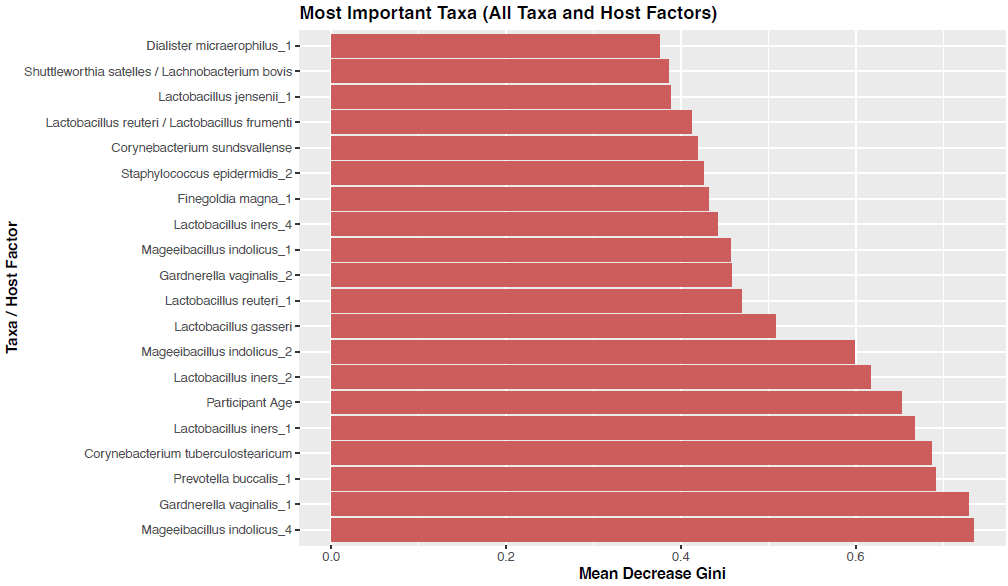

Supplement: 1 [file NIHMS2186768-supplement-1.docx]
